# 1230. Evaluation of the Interplay Between β-lactamases and Porin Production on β-Lactam MICs in *Klebsiella pneumoniae*

**DOI:** 10.1093/ofid/ofab466.1422

**Published:** 2021-12-04

**Authors:** Alyssa K Whitney, Nancy D Hanson

**Affiliations:** 1 Creighton University, Omaha, Nebraska; 2 Creighton University/Dept of Medical Microbiology and Immunology, Omaha, Nebraska

## Abstract

**Background:**

*K. pneumoniae* can emerge resistant to β-lactam antibiotics through the production of β-lactamase enzymes and/or loss of the outer membrane porins, OmpK35, OmpK36, and/or PhoE. While both mechanisms are hypothesized to work synergistically, β-lactamases have been the focus of previous studies. As a result, the contribution of outer membrane porin loss to the β-lactam minimum inhibitory concentration (MIC) is unknown. The objective of this study was to evaluate the contribution of specific β-lactamases and porin production to β-lactam susceptibility. We hypothesize that production of a β-lactamase in a clinical isolate deficient in 3 major porins will result in higher β-lactam MICs but not always a resistant phenotype.

**Methods:**

The structural gene and promoter of CTX-M-14, CTX-M-15, and CMY-2 were cloned into a low copy number vector and transformed into Kp 23, a wild-type clinical isolate, and KPM 20, a clinical isolate deficient in OmpK35/36 and PhoE. MICs to ceftolozane/tazobactam, cefotaxime, ceftazidime, cefepime, and meropenem were determined by E-test. Kp 23 and KPM 20 were characterized by Western blot and whole genome sequencing.

**Results:**

Production of CMY-2 alone led to a resistant phenotype for ceftolozane/tazobactam, cefotaxime, and ceftazidime regardless of porin production (Figure 1). CMY-2 production in KPM 20 resulted in non-susceptibility to meropenem. Both clones were susceptible to cefepime. Production of CTX-M-14 and CTX-M-15 in Kp 23 resulted in only cefotaxime resistance. Production of CTX-M-14 and CTX-M-15 in KPM 20 resulted in isolates non-susceptible to all antibiotics tested.

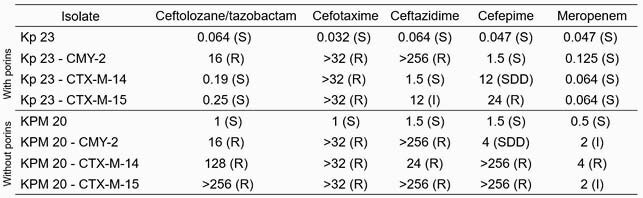

Figure 1. MICs of K. pneumoniae clones against panel of β-lactam antibiotics.

**Conclusion:**

When evaluating clinical isolates, it is impossible to determine the contribution of individual resistance mechanisms in the susceptibility pattern. This study demonstrated that resistance is not solely dependent on the β-lactamase produced and that the impact of porin deficiency varies with the antibiotic being evaluated. These data suggest that antibiotic selection may be more nuanced and that a broader range of therapeutics may be available given the appropriate diagnostic tools. Understanding the contributions of all resistance mechanisms is necessary to inform selection of the most appropriate antibiotic therapy.

**Disclosures:**

**Nancy D. Hanson, PhD**, **Merck** (Grant/Research Support)

